# Lactylation in Glioblastoma: A Novel Epigenetic Modifier Bridging Epigenetic Plasticity and Metabolic Reprogramming

**DOI:** 10.3390/ijms26073368

**Published:** 2025-04-04

**Authors:** Qingya Qiu, Hui Deng, Ping Song, Yushu Liu, Mengxian Zhang

**Affiliations:** Department of Oncology, Tongji Hospital, Tongji Medical College, Huazhong University of Science and Technology, Wuhan 430030, China; 15271987716@163.com (Q.Q.); d202382343@hust.edu.cn (H.D.); songping202212@163.com (P.S.); liuyushu9912@163.com (Y.L.)

**Keywords:** glioblastoma, lactylation, epigenetic remodeling, metabolic reprogramming

## Abstract

Glioblastoma, the most common and aggressive primary malignant brain tumor, is characterized by a high rate of recurrence, disability, and lethality. Therefore, there is a pressing need to develop more effective prognostic biomarkers and treatment approaches for glioblastoma. Lactylation, an emerging form of protein post-translational modification, has been closely associated with lactate, a metabolite of glycolysis. Since the initial identification of lactylation sites in core histones in 2019, accumulating evidence has shown the critical role that lactylation plays in glioblastoma development, assessment of poor clinical prognosis, and immunosuppression, which provides a fresh angle for investigating the connection between metabolic reprogramming and epigenetic plasticity in glioblastoma cells. The objective of this paper is to present an overview of the metabolic and epigenetic roles of lactylation in the expanding field of glioblastoma research and explore the practical value of developing novel treatment plans combining targeted therapy and immunotherapy.

## 1. Introduction

The latest statistics published in the Global Cancer Observatory database indicate a significant increase in the morbidity and mortality of malignant tumors in recent years, making them an urgent public health concern [1]. Glioblastoma (GBM), the most lethal primary tumor of the central nervous system in adults, accounts for approximately 80% of all primary malignant cerebral tumors. Moreover, it is the main oncological trigger of death among young adult males, with a median survival of typically less than 15 months and a 5-year survival rate of less than 5% [2,3,4]. Nowadays, surgery, radiotherapy, and chemotherapy are the main treatments for GBM [5,6]. However, little notable information on therapeutic efficacy has been observed thus far. The 2021 WHO Classification of Central Nervous System Tumors underscores that the development of molecular pathology stratification will facilitate more precise prognostic assessment, diagnosis, and treatment of GBM [7]. Nevertheless, the current lack of specific biomarkers poses significant challenges to the development of targeted therapies for GBM [8,9]. With the emergence of new insights from biomedical research, lactate, long regarded solely as a byproduct of glycolytic, is increasingly becoming more widely acknowledged for its function in regulating gene expression and cellular signaling [10]. As knowledge of cancer mechanisms has progressed, cancer-related metabolic disorders can contribute to tumorigenesis by inducing epigenetic changes [11]. Notably, there has been a recent surge of interest in lactylation, a novel protein post-translational modification (PTM) induced by lactate, which has been observed in the malignant progression of several tumors, including hepatocellular carcinoma, pancreatic ductal adenocarcinoma, and melanoma [12,13,14]. The metabolic rewiring of glycolysis in GBM cells has been observed to cause epigenetic remodeling associated with lactylation, which in turn leads to the signaling pathways affecting various biological processes in GBM. Targeting lactate metabolism and lactylation pathway, as demonstrated in references [15,16], has shown promising results. Consequently, to improve the therapeutic efficacy of GBM, it is of great significance to explore in depth the mechanisms of tumorigenesis, as well as identifying new biomarkers, therapeutic targets, and individualized treatment regimens for GBM.

## 2. Glycolytic Metabolic Reprogramming in Glioblastoma

Metabolic rewiring and non-mutational epigenetic reprogramming are distinctive hallmarks of cancer [17]. In contrast to the irreversible nature of genetic mutations, non-mutational epigenetic modifications represent a dynamic and reversible process that regulates gene expression primarily through a range of mechanisms, including DNA methylation regulating gene expression, histone modifications affecting chromatin structure, and non-coding RNA interactions with target mRNA [18]. Among these mechanisms, protein PTMs impact the structure and function of proteins via the addition of chemical groups to amino acid residues of precursor proteins, including methylation, acetylation, phosphorylation, glycosylation, and the newly discovered lactylation [19]. Epidemiological studies suggest that metabolic reprogramming not only supplies energy for aberrant tumor proliferation but also plays a critical regulatory role in the epigenetic modification of tumors. Most of the substrates of PTMs derive from intracellular metabolic processes. Metabolites like acetyl-coenzyme A, S-adenosylmethionine, and lactate are involved in the regulation of acetylation, methylation, lactate, and other PTMs via the corresponding chromatin-writing enzymes, driving the reprogramming of the epigenomic landscape [20,21,22]. In recent years, there has been increasing evidence that metabolic remodeling in GBM is highly dependent on the glycolytic pathway, with lactate as a critical product of this pathway affecting a wide range of biological functions in tumors, thereby increasing the oncogenic burden.

### 2.1. Lactate Accumulation

First described by Otto Warburg in 1923, the Warburg effect refers to the metabolic reprogramming of cancer cells to prioritize glycolysis for lactate production, even under oxygen-replete conditions, contrasting with normal cells that typically transiently activate glycolysis to produce lactate during hypoxic stress [23,24]. This phenomenon is especially pronounced in glioblastoma; likewise, glutamine catabolism also provides glioblastoma cells with a significant source of NADPH, resulting in the majority of glutamine-derived carbon being secreted as lactate [25]. The distinct hallmarks of cancer cell metabolism are considered to provide valuable insights for directing tumor therapy [26], and yet, the mechanisms of the metabolic lactate in GBM remain incompletely understood. It is acknowledged that cell proliferation is associated with multiple signaling pathways regulated by glycolytic metabolism [27]. Transcription factors, including hypoxia-inducible factor 1 alpha (HIF-1α), c-MYC, and p53, are known to be primarily responsible for establishing the glycolytic phenotype in cancer. For instance, the mammalian targets of rapamycin (mTOR) signaling have the potential to promote glycolysis by activating glucose transporter 1 (GLUT1) expression through the transcription factors HIF1α and MYC [28]. Additionally, expression of the p53-inducible gene, the glycolytic and apoptotic regulator (TIGAR), reduces glycolytic intermediates within cells, thereby inhibiting the glycolytic pathways and protecting cells from apoptosis [29]. In general, dysregulation of pivotal transcription factors in GBM, particularly TGF-β1-mediated upregulation of PFKFB3 [30] and mTORC2-dependent activation of c-Myc [31], collectively drives glycolytic reprogramming in GBM cells; that is, regardless of the hypoxic conditions, these cells preferentially utilize glycolysis for energy production and biosynthetic precursor synthesis for aberrant proliferation. Consequently, GBM exhibits characteristic metabolic profiles marked by lower glucose concentrations and elevated lactate accumulation compared to normal tissue [32]. The resultant lactate enrichment within the tumor microenvironment (TME) can subsequently promote gliomagenesis [33]. This phenomenon indicates that therapeutic strategies aimed at targeting the Warburg effect may offer viable applications in the management of GBM.

### 2.2. Lactate Transport

Intracellular and extracellular lactate shuttle functions as the link between glycolysis and downstream signaling pathways [34]. Lactate generated by glycolysis in the tumor cytoplasm undergoes regulated transport through specific receptors on the cell surface, primarily monocarboxylate transporters (MCTs), G-protein-coupled receptors (GPCRs), and hydroxycarboxylic acid receptor 1 (HCAR1) [35]. Specifically, the exchange of intracellular and extracellular lactate is mainly mediated by MCT1 and MCT4. MCT1 primarily mediates the uptake of lactate, whereas MCT4 mainly facilitates the export of intracellular lactate. The direction of facilitated transport is primarily driven by the concentration gradient; consequently, elevated lactate accumulation in GBM cells and the TME critically regulates intercellular lactate shuttle [36,37]. It has been established that elevated levels of lactate in GBM can stimulate the expression of MCT1 and HCAR1 to promote the proliferation of tumor cells. Furthermore, lactate directly enhances the expression of GPR81, a member of the GPCR family, in endothelial cells in an MCT1-dependent manner, thereby mediating the activation of multiple intracellular signaling pathways [38,39,40]. Accordingly, it can be speculated that interfering with lactate transport by inhibiting the expression of MCTs and HCAR1 in tumor cells may represent an effective strategy to impede tumor growth.

### 2.3. The Biological Role of Lactate in GBM

It is noteworthy that research on lactate in tumors is progressing rapidly. Karin Fischer et al. demonstrate that serum lactate levels in patients with lymphoma, breast cancer, melanoma, and other cancers exhibit a positive correlation with tumor burden [41]. In addition to tumor cells, the catabolic host cells comprising the TME, including cancer-associated fibroblasts (CAFs), vascular endothelial cells, tumor-associated macrophages (TAMs), and regulatory T cells (Tregs), can also produce substantial amounts of lactate through induction of glycolysis. Substantial amounts of lactate are then absorbed from the TME into the cell via MCT1 to provide high-energy fuel for cancer cells and can also be released into the extracellular environment via MCT4 to prevent cellular acidosis [42,43,44]. In recent years, an increasing number of studies have elucidated that the Warburg effect and the lactate shuttle effect facilitate interactions between glioblastoma cells and the TME, leading to the formation of a metabolic network that creates an acidic microenvironment conducive to tumor cell survival. Moreover, this environment can result in immunosuppression by influencing cellular metabolism and signaling, inhibiting the function of T lymphocytes, thereby providing tumor cells with an advantage to evade immune surveillance, which promotes cancer cell proliferation and metastasis [45,46,47].

Lactate acts not only as a carbon source that provides energy to tumor cells during glycolysis [48] but also as a key signaling molecule that influences transcriptional regulation and epigenetic modifications in GBM, thereby regulating a wide range of biological functions in tumors [49]. Previous studies have shown that lactate can promote M2-like polarization of TAMs by upregulating HIF1α-mediated expression of M2-associated genes, including vascular endothelial growth factor (VEGF) and arginase 1 (Arg1), thereby facilitating tumor progression through activation of critical signal transduction pathways [50]. Moreover, Liu et al. found that lactate transmits signals that inhibit the SUMO protease SENP1, thereby reshaping the anaphase-promoting complex (APC/C) to stimulate the timed degradation of cell cycle proteins, which modulates tumor cell division and proliferation [51]. Furthermore, it has also been shown that elevated lactate levels can drive GBM progression by enhancing cell survival [52] and induce NDRG3 protein expression, subsequently activating the Raf–ERK pathway to promote tumor angiogenesis [53]. This robust neovascularization contributes to the adaptation of tumor to rapid growth and metastasis. Specific deletion of promoter methylation of glycolytic genes, particularly lactate dehydrogenase A (LDHA), results in the acquisition of a hyperglycolytic phenotype [54], leading to lactate accumulation that correlates with the grade of glioma, enhanced proliferative invasiveness, and poorer survival [36,55,56]. In summary, the lactate level in GBM serves as an indicator for prognosticating tumor outcomes. Equally importantly, targeting the lactate metabolic pathways spurs the development of an effective treatment for GBM.

## 3. Lactylation Landscape Bridges Metabolic Reprogramming and Epigenetic Plasticity

**Figure 1:**
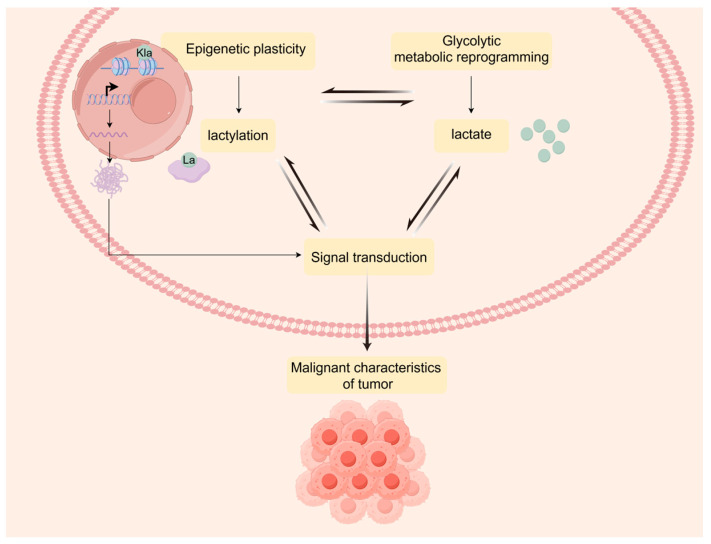
Metabolic and epigenetic interactions through lactylation. Glycolytic metabolic reprogramming and epigenetic plasticity associated with lactylation enable information interchange via lactate and trigger corresponding signal transduction to promote the malignant characteristics of the tumor.

In 2019, Zhang et al. [57] employed mass spectrometry to report histone lactylation for the first time, revealing an enzyme-catalyzed process, wherein lactate serves as a substrate to transfer a lactoyl group to a protein lysine residue, thereby opening the door to further investigation into lactylation. Lactate can covalently bind to histones and other pivotal proteins, thereby affecting their conformation and functional activity. As a rising star in the field of epigenetics, lactylation is not only associated with the metabolic reprogramming of cancers to produce lactate, but it also enhances the transcription of signaling pathways that promote tumor progression, thus participating in driving the phenotypic transformation of cells, enabling tumors to rapidly adapt to environmental changes [58,59]. Subsequently, this progress can provide valuable biomarkers for tumor prognostic evaluation and therapeutic response assessment. This is illustrated in Figure 1.

### 3.1. The Regulatory Mechanisms of Lactylation

As with other PTMs, lactylation can be regulated enzymatically through specific enzymes involved in the deposition of lactate (writers), its removal (erasers), and its recognition (readers). However, emerging evidence also suggests that non-enzymatic mechanisms may contribute to lactylation dynamics, particularly in non-histone contexts. Nevertheless, the precise enzymatic mechanisms underlying lactylation remain poorly understood. Lactylation on histones in the nucleosome is associated with intracellular lactate levels. The available reports suggest that the protein p300 functions as the writer protein for lactylation, catalyzing the transfer of lactyl-coenzyme A (lactyl-CoA) produced from lactate as a substrate to the lysine residues of proteins. The triggering of lactylation modification results in alterations to the structure of histones. Following this modification, lactylated histones are specifically recognized and bound by the corresponding effector proteins of the lactylated recognition enzymes (readers). This affects gene expression in tumor and immune cells, thus further regulating tumor progression and the immunosuppression effect [60]. Additionally, histone deacetylase (HDAC) also acts as histone delactylase, markedly reducing the extent of lactylation. In this respect, HDAC1-3 and SIRT1-3 are highly effective delactylases capable of reversing histone lactylation [61,62]. SIRT2 has been identified as a histone eraser in neuroblastoma that reduces lactylation modification and, as a result, inhibits proliferation and migration [63]. This evidence implies that histone lactylation is a dynamic and reversible process, installed and removed by regulatory enzymes. Therefore, it can be concluded that these key enzymes offer new possibilities for targeted therapy of GBM. Notably, sirtuins have isozyme-specific roles in GBM pathogenesis; for example, SIRT1 promotes tumor proliferation via c-Myc activation and metabolic reprogramming [64], while SIRT3 dynamically regulates lactylation under hypoxia to restore oxidative phosphorylation (OXPHOS) and interacts with TRAP1 to sustain ROS scavenging in glioma stem cells [65]. These dual roles underscore the paradoxical therapeutic potential of targeting sirtuins in GBM, necessitating a precise balance between sirtuin-mediated lactylation-dependent epigenetic regulation and metabolic adaptation in therapeutic strategies.

Non-histone lactylation was initially detected through liquid chromatography–tandem mass spectrometry (LC-MS/MS) analysis of lactylation in Staphylococcus griseus [66]. Subsequently, an ever-growing number of studies have revealed the broad promise of lactylation, extending beyond the scope of histones. In a 2020 study, Gaffney et al. [67] proposed that lactylation also represents a non-enzymatic lactylation pathway utilizing lactoyl-glutathione (LGSH) as a substrate. Moreover, lactylation affects metabolic enzymes in the glycolytic pathway. In particular, alanyl-tRNA synthetases AARS1 and AARS2 play the role of lactyltransferases by binding lactate to induce lysine lactylation of non-histone proteins. Notably, depletion of AARS1 and AARS2 results in the nearly complete erasure of lactylation induced by lactate [68]. Furthermore, Wan et al. also revealed a broad prospect beyond lactylation on histones, which form the existing unenriched human proteome resources, thereby enhancing protein diversity [69]. In short, the discovery of non-histone lactylation provides further evidence of the importance of lactylation in regulating the activity of numerous enzymes and signaling pathways, supporting its role as a biomarker.

### 3.2. Detection Methods for Lactylation

To date, several techniques are available for the detection of lactylation, among which metabolic tracing with stable carbon isotope-labeled lactate can demonstrate the involvement of lactate in histone lactylation. Additionally, 13C NMR spectroscopy can be employed to observe glucose and glutamine metabolism in GBM, thereby providing insights into how these nutrients are utilized by cancer cells for growth [25]. Furthermore, mass spectrometry (MS) is a highly sensitive and powerful analytical technique capable of the detection and quantification of molecules in tissues. The metabolism of glucose is an attractive target for investigation using magnetic resonance spectroscopy (MRS) imaging, a non-invasive technique for detecting brain tumors based on NMR technology [70,71]. This approach offers potential for qualitative diagnosis, as well as for assessing the prognosis in GBM. However, further substantiation is required to fully validate its use in the diagnosis of malignancy in GBM [72,73]. While tandem mass spectrometry (MS/MS) is better suited for the quantification of individual metabolic compounds in tissues, high-resolution liquid chromatography (HPLC)-MS/MS offers greater convenience and accuracy [74]. Currently, the antibodies targeting lactylation have been well established, including pan-specific antibodies recognizing lactylated lysine residues across all proteins (anti-pan-Kla) and site-specific antibodies such as anti-H3K18la, anti-H3K14la, and anti-XRCC1 lac247. These antibodies have been rigorously validated via mass spectrometry and are applicable to multiple experimental approaches, including Western blot, immunofluorescence, and chromatin immunoprecipitation sequencing (ChIP-seq). Their utility has been demonstrated in diverse cancer models, particularly GBM, bladder cancer, and melanoma, providing critical tools for exploring lactylation-driven epigenetic regulation in tumor [14,75,76,77]. Nevertheless, the number of potential lactylation sites is considerable, and current antibodies may not specifically recognize all types of sites. The experimental techniques like Western blot and immunohistochemistry for identifying them are both costly and time-consuming. In order to predict lactylation sites in a more convenient and efficient manner, Jiang et al. [78] pioneered the construction of the inaugural lysine lactylation benchmark dataset and designed FSL-Kla, a predictor for calculating and predicting lactylation sites. Each of these methods possesses distinctive advantages and disadvantages, and it is still a challenge to identify specific targets and overcome off-target effects for lactylation. The advancement of experiments and technology is essential to facilitate the development of accurate and cost-effective standardized methods.

### 3.3. Metabolic Interaction of Lactylation in Glioblastoma

**Figure 2:**
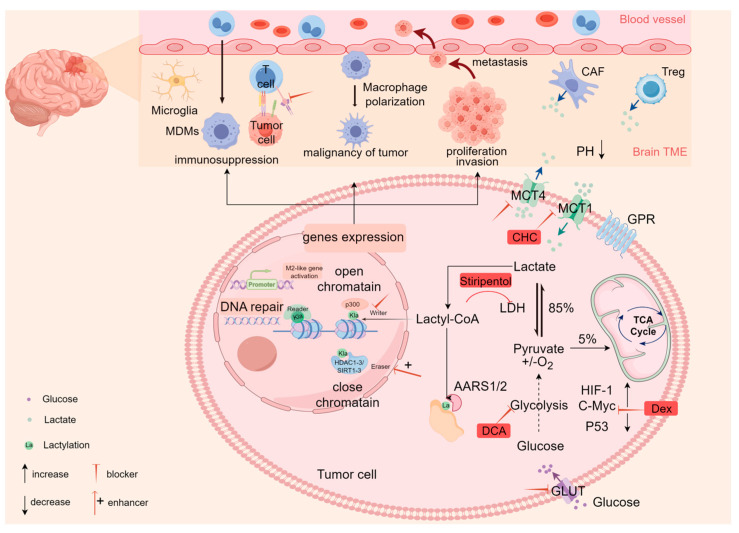
Role of lactate and lactylation in GBM. This schematic delineates the lactate–lactylation regulatory network. The glycolytic phenotype, driven by HIF-1α and c-Myc and restrained by p53, regulates lactate production, which is exported via MCT4 to acidify TME, fostering malignant progression. Intracellular lactate, derived from both endogenous glycolysis and MCT1-mediated uptake, induces histone and non-histone lactylation, regulating gene expression and signaling pathways, further driving tumor invasion, metastasis, and immunosuppression. The current targeting strategies mainly target lactate production (e.g., DCA, Stiripentol) and transport (MCT inhibitors), which are highlighted in red in the figure, providing potential interventions to disrupt this epigenetic–metabolic network in GBM. Abbreviations: HIF-1, hypoxia-inducible factor 1; GLUT, glucose transporter ; TME, tumor microenvironment; MCT1, monocarboxylate transporter 1; MCT4, monocarboxylate transporter 4; GPR, G-protein-coupled receptor; CAF, cancer-associated fibroblast; Treg, regulatory T cells; DCA, dichloroacetate; Dex, dexmedetomidine; lactyl-CoA, lactyl-coenzyme A; MDMs, monocyte-derived macrophages; CHC, α-cyano-4-hydroxycinnamate.

Histone lactylation represents a crucial nexus for glycolytic metabolic reprogramming and epigenetic modifications. The transcription factor Glis1 closes chromatin at somatic genes to upregulate glycolysis and enhance cellular lactate levels, thereby enhancing locus lactylation for cellular reprogramming. This process reveals a metabolome–epigenome–metabolome signaling cascade response [79]. GBM typically exhibits glycolytic metabolic reprogramming, resulting in the accumulation of lactate, which provides a rich substrate for lactylation. Histone lactylation can profoundly affect gene transcription in chromatin. Furthermore, the enzymes involved in energy metabolism or signal transduction may also be more susceptible to undergoing lactylation modification. Both processes can influence a variety of metabolic processes associated with tumor cell proliferation, migration, and invasion [80,81]. The mechanism of lactylation, linking metabolic reprogramming to epigenetic plasticity, is depicted in Figure 2.

#### 3.3.1. Lactylation Promotes GBM Cell Proliferation

GBM is a highly aggressive tumor that typically manifests rapid cell proliferation and remarkable cell viability. In recent years, the effects of lactylation on GBM proliferation and apoptosis have gradually attracted increasing scientific attention. Li et al. found that the NF-κB signaling pathway drives Warburg-effect-induced histone lactylation and promotes the expression of the long-chain non-coding RNA LINC01127, which in turn promotes the GBM cells’ self-renewal and tumor progression via the MAP4K4/JNK axis [82]. Furthermore, histone lactylation has been reported to upregulate the expression of the human-derived nucleic acid alkylation damage repair enzyme (ALKBH3) while simultaneously decreasing the formation of the tumor suppressor promyelocytic leukemia protein (PML) condensate. These changes have been demonstrated to promote the malignant transformation of cancer [83]. Under hypoxic conditions, the accumulation of lactate in glioma cells is taken up by macrophages via MCT1, which upregulates histone H3K18 lactylation and regulates the expression of TNFSF9. This process induces M2 polarization in a histone-lactylation-dependent manner, thereby promoting the malignant progression of gliomas [84]. The lactylation-associated pathways represent a promising avenue for further research into the regulation of GBM proliferation. It is anticipated that scientists will elucidate the underlying mechanisms in greater detail, potentially leading to the identification of novel therapeutic targets.

#### 3.3.2. The Effect of Lactylation on the TME of GBM

In the tumor immune microenvironment of GBM, lactylation plays a significant role in regulating inflammatory responses, promoting tumor invasion and metastasis, and modulating immune responses. Research has identified lactylation mechanisms in other tissues. Specifically, the epigenetic reprogramming of renal tubular glycolysis, which is mediated by the key glycolytic enzyme PFKFB3, allows lactate to significantly enhance histone H4K12 lactylation. This phenomenon is enriched at the promoters of NF-κB signaling genes, which subsequently activates transcription and promotes an inflammatory response, thus driving the process of epithelial–mesenchymal transition (EMT) [85,86]. This evidence indicates that lactylation may have a potential impact on the GBM inflammatory response. In the GBM microenvironment, immunosuppressive M1 macrophages with high glycolytic activity are capable of utilizing the glycolytic product lactate as an epigenetic regulator and promote the upregulation of the expression of M2-like genes during the polarization of M1 macrophages, thereby influencing gene expression and driving immune escape [87]. Moreover, Leo et al. emphasized the significance of histone lactylation in enhancing the immunosuppressive capacity of monocyte-derived macrophage MDMs via PERK-driven glucose metabolism in the GBM [88]. Likewise, lactate has been demonstrated to inhibit cGAMP production by mediating lactylation of cGAS, which also leads to a reduction in natural immune surveillance capacity in patients with GBM [89]. Notably, histone lactylation labeling has been observed to promote CD8 T-cell transcriptional activation and proliferation [90]. Moreover, the metabolic and epigenetic status of Th17 cells is also regulated by lactate. Increased levels of H3K18la reprogram the pro-inflammatory T-cell phenotype to Tregs [91]. Lactate can also promote the modulation of Lys72 lactylation in MOESIN to enhance the stability and immunosuppressive capacity of Tregs, thereby facilitating tumor progression [92]. Collectively, elevated lactate levels in TME are crucial for lactylation and suggest that histone lactylation in tumor cells, immune cells, and stromal cells may be markedly enhanced in glioblastoma TME, which may regulate immune cell function and alter the TME, thus playing a pivotal role in tumor cell invasion and immune evasion through the modulation of multiple signaling pathways.

## 4. The Clinical Application Prospect of Lactylation in GBM

GBM is categorized as a “cold tumor”, with a generally poor prognosis for patients. With an in-depth understanding of the characteristics of metabolic reprogramming and epigenetic plasticity in tumors, the clinical application prospect of the glycolysis product lactate and its associated modifications in GBM has sparked intense discussion. Lactate and lactylation have been found to exert distinct regulatory effects on multiple aspects of tumorigenesis, immunosuppression, and therapeutic resistance [93,94], which may be potential biomarkers, provide potential targets for GBM, and enhance the efficacy of anti-tumor drugs.

### 4.1. Chemoradiotherapy Resistance and Prognostic Biomarkers

Over the last few years, there has been growing evidence that lactate and lactylation levels are significantly higher in GBM than in normal tissues, which are often strongly associated with biological characteristics, treatment resistance, and shorter patient survival of glioblastoma. Consequently, early monitoring of lactate and lactylation levels may assist in predicting patient survival and tumor progression [54]. The relationship between lactylation and resistance to chemoradiotherapy is currently under investigation. Chen et al. have found that TIP60-mediated NBS1 lactylation is crucial for effective DNA repair and contributes to chemotherapy resistance in gastric cancer. Additionally, they established that elevated NBS1 lactylation level is indicative of a poor prognosis for chemotherapy in patients [95]. In this respect, the DNA repair protein MRE11 demonstrates significant lactylation levels that positively correlate with lactate concentration. Lactylated MRE11 enhances its DNA-binding capacity to facilitate DNA end resection, thereby driving excessive activation of homologous recombination (HR)-mediated DNA repair. This hyperactive HR pathway consequently reduces tumor cell sensitivity to specific chemotherapeutic agents (e.g., temozolomide) and radiotherapy [96,97]. Furthermore, Yue et al. observed that lactylation was upregulated in recurrent GBM tissues, which in turn inhibited mismatch repair (MMR) [75]. Moreover, through immunoblot analysis, it was found that the interaction between ALDH1A3, the aldehyde dehydrogenase isoform, and PKM2 promotes the production of lactate in GBM cells, which in turn leads to lactylation of XRCC1 at the K247 locus, a scaffolding protein to repair DNA, regulating the cellular localization of transcription factors and the ability of XRCC1 to bind to DNA. This modification enhances DNA damage repair, enhancing the tolerance of GBM to DNA damage during radiotherapy and temozolomide (TMZ) [76,98]. GTPSCS interacts with p300 to form a functional lactoyltransferase complex in vivo, which enhances histone lactylation levels, modulates GDF15 expression, and ultimately confers radioresistance in gliomas [99]. Furthermore, lactate production and lactylation are regarded to influence the host’s anti-tumor immune response by promoting the polarization of TAMs [94]. Consequently, it is hypothesized that lactylation may be a poor prognostic factor for GBM immunosuppression and a predictor of radiotherapy and chemotherapy sensitivity.

### 4.2. Unraveling Therapeutic Targets and Strategies

The current treatment strategy for GBM mainly involves maximal resection within a safe range, supplemented by a combination of chemotherapy, radiotherapy, and electric field therapy. Although the extent of resection correlates with the efficacy of the treatment, complete resection of GBM is extremely challenging due to the unique anatomical structure of the tumor [100,101]. GBM exhibits remarkable tumor heterogeneity, robust metabolism, and nucleotide regeneration [102], which collectively contribute to its high invasive and immune escape ability, rendering the tumor significantly resistant to existing treatment options, thereby leading to recurrence being almost inevitable, and patients continue to experience high rates of mortality [103,104]. While targeted therapy and immunotherapy are flourishing and have brought new hope to this filed, studies focusing on lactate and lactylation targets have yielded positive outcomes in the preliminary exploration of relevant tumors. Consequently, further research in this field will provide new possibilities for enhancing the outcomes of GBM patients.

#### 4.2.1. Targeting Lactate Metabolism

Lactylation connects metabolic reprogramming to generegulation, thereby promoting oncogenic signals pathways, which provides new insights into the potential for precision targeting of tumorigenic mechanisms. A reduction in lactate production achieved through the re-regulation of glycolytic pathways can reduce the levels of lactylation, a process that suggests multiple potential targets for cancer [105]. The existing studies have mainly focused on targeting lactate metabolism. Zhou et al. revealed the miR-365–HOXA9–HIF-1α regulatory axis, which promotes glycolysis in cutaneous squamous cell carcinoma (cSCC) and provides a potential target for intervention in cSCC therapy [106]. Meanwhile, high levels of GLUT3 in glioblastomas selectively mediate glucose uptake, indicating that it confers a substantial growth-competitive advantage to cancer cells, which may provide a potential therapeutic window for targeting GLUT3 to reduce the production of lactate caused by glycolysis [107]. Furthermore, numerous preclinical studies have revealed that inhibition of LDHA can markedly enhance the antiproliferative effects in a range of cancer cells, including those of the breast, prostate, and pancreas [108]. In particular, silencing LDHA could inhibit cell proliferation and enhance the chemosensitivity of glioma cells to temozolomide [109]. The use of DCA and oxalate has shown efficacy in reducing lactate production by modulating the activity of the key enzymes of glycolysis, pyruvate dehydrogenase (PDH) and lactate dehydrogenase (LDH), respectively, which can reduce lysine lactylation, and it has shown promising efficacy in glioblastoma [110,111]. Moreover, the silencing of BMAL1 has been found to inhibit glycolysis, thereby promoting M1 polarization via the LDHA/lactate axis, further inhibiting M2 polarization and angiogenesis in GBM cells, and to sensitize GBM cells to bevacizumab [112]. ChIP-Seq coupled with systemic metabolite profiling in GBM models revealed that the suppression of c-Myc, a pro-survival factor and a major regulator of glycolysis, can be achieved with HDAC inhibitors (panobinostat, vorinostat, and romidepsin), which have been discovered to reduce glycolysis in GBM by reducing c-Myc protein levels and improve survival rates in GBM-bearing animals [113,114]. Finally, with the advent of genetic engineering technology, microRNAs have been identified as inhibitors of mTORC2 and c-Myc. The CRISPR/Cas9 technology has opened up a new avenue for the regulation of glycolytic metabolism in GBM [115]. In addition to influencing the level of lactylation, inhibitors of lactate metabolism may exert broader and more intricate effects on cellular metabolic responses. Consequently, future research must address the critical issue of enhancing selectivity and reducing the adverse effects of these inhibitors when developing targeted therapies.

#### 4.2.2. Targeting Lactate Transport

Drugs targeting MCT1 and MCT4 are currently undergoing preclinical studies. The research progress on the therapeutic outcome of small molecule inhibitors of lactate-associated transport proteins (MCTs) is encouraging in glioblastoma [116]. In GBM, the glycolytic metabolite lactate is taken up by macrophages via MCT1, thereby inducing M2 polarization. It is worth noting that this process can be reversed by silencing the MCT1; that is to say, blocking the transport function of MCT1 can reduce the entry of lactate into macrophages, thereby inhibiting the lactylation process and restoring innate immune surveillance, inducing cancer cell apoptosis [90]. Studies have shown that the MCT1 inhibitor α-cyano-4-hydroxycinnamate (CHC) exhibits anti-tumor and anti-angiogenic activity in gliomas and enhances the efficacy of temozolomide [117]. Furthermore, MCT4 inhibitors can effectively block lactate efflux into the TME, thereby inhibiting the formation of an acidic microenvironment and weakening the promotional effect of this microenvironment on tumor growth [118].

#### 4.2.3. Targeting Lactylation

Epigenetic mechanisms can be regarded as a dynamic and reversible process. Consequently, there is a heightened interest in the molecular mechanisms associated with tumor epigenetic plasticity for tumor-targeted therapies [119]. Lactylation-associated epigenetic modulators, such as writers, readers, and erasers, represent a promising avenue for targeted intervention but have not been fully investigated; research on them remains in the preclinical stage, with no large-scale clinical trials conducted to date. Meanwhile, the overall metabolic regulation network of lactylation holds multiple potential drug targets, several of which have been verified in GBM preclinical models. LINC01127 knockout or JNK inhibition (SP600125) attenuates H3 histone-lactylation-dependent signaling, prolonging survival in orthotopic GBM models [82]. HIF-1α promotes YTHDF2 upregulation via H3K18 lactylation, activating BNIP3-mediated mitophagy and GBM proliferation and invasiveness, while YTHDF2 knockdown reverses these effects [120]. Similarly, Liu et al. demonstrated with ChIP-seq that targeting GDF15 inhibited GTPSCS/p300/H3K18la axis activation, significantly attenuating glioma progression [98]. Of note, it was identified that a clinical antiepileptic drug, stiripentol, can penetrate the blood–brain barrier, inhibit the activity of LDH, and markedly reduce H3K9la levels, which makes GBM cells more sensitive to TMZ. Combined with TMZ, it overcomes chemoresistance and prolongs survival in murine models [75]. Meanwhile, knockout of ALDHA3 in patient-derived GSCs using CRISPR-Cas9, as well as small molecule D34-919, disrupts ALDH1A3–PKM2 interaction, suppresses XRCC1 lactylation, and enhances the sensitivity of radiotherapy and chemotherapy. Treatment with D34-919 in combination with TMZ or radiotherapy shows superior efficacy compared to each drug or radiotherapy alone, which was validated not only in murine GBM models but also in patient-derived GBM organoids [76]. These findings not only shed light on the mechanism of lactylation in GBM TMZ resistance but also provide a novel insight into the potential of combination therapy to improve the prognosis of GBM. Furthermore, dex inhibits c-Myc lactylation and stability, impairing GBM proliferation and metastasis [121]. The clinical translatability of repurposed agents warrants further investigation.

Moreover, lactylation affects not only the biological characteristics of tumors but also participates in the regulation of the TME, particularly with regard to immune evasion [122]. Glycolysis-active GBM cells drive PERK-mediated histone lactylation in MDMs, upregulating IL-10 to foster immunosuppression. Targeting PERK in combination with immunotherapy improves the efficacy of immunotherapy for GBM [88]. CBX3 knockdown reduces histone lactylation in glioma stem cells, enhancing immunotherapy sensitivity [123]. GBM models show that oxamate, an LDH inhibitor, reduces H3K18 lactylation to downregulate CD73 and CCR8 expression, enhancing CAR-T cell efficacy against GBM by altering the phenotype of immune molecules in the TME and increasing the infiltration of CAR-T regulatory T cells in GBM [124]. Importantly, lower levels of lactylation in Treg cells have been shown to enhance the sensitivity of PD-1 antibodies [92]. Broader implications exist across other tumors. MRE11 K673 lactylation promotes chemoresistance via homologous recombination repair. Targeted inhibition using cell-penetrating peptide (e.g., K673 peptide) or LDHI significantly enhances chemosensitivity in colorectal cancer [90]. The outcome of combining the LDHI and PD-1 antibodies is more pronounced than that of a PD-1 antibody alone.

Collectively, as part of a multipronged strategy, targeting the lactylation network represents a promising paradigm to disrupt oncogenic signaling pathways, reprogram the TME, and overcome therapeutic resistance in GBM.

In light of the intratumoral heterogeneity and the complexity of the metabolic landscape in GBM, regulating lactate metabolism alone may not be sufficient to achieve significant anti-tumor effects. Moreover, strategies targeting lactate homeostasis have thus far demonstrated only limited success in clinical applications. However, reprogramming lactate production by targeting glycolytic metabolism or lactylation-related pathways is anticipated to augment the efficacy of immunotherapy. Consequently, combining this innovative therapy with chemotherapy, radiotherapy, and immunotherapy is expected to yield a superior integrated therapeutic effect. Additionally, emerging evidence highlights metabolic–epigenetic cross-talk among lysine acylations (e.g., acetylation, crotonylation, butyrylation, lactylation) and methylation, which collectively regulate gene expression and cellular adaptation [125]. Notably, they share overlapping regulatory machinery involving common writers (e.g., p300/CBP acetyltransferases) and erasers (e.g., HDAC1-3 and SIRT1-3 deacetylases), which dynamically balance the modification states in response to metabolic and epigenetic cues [64,126]. Lactylation uniquely competes with acetylation under hypoxia by inhibiting HDACs, indirectly elevating acetylation levels [127]. Dai et al. demonstrated that lactylation and crotonylation regulate cell proliferation by cooperatively remodeling histone acylations and orchestrating gene expression [128]. These offer therapeutic target opportunities for metabolic–epigenetic diseases and allow better development of personalized and comprehensive treatment options for GBM.

## 5. Decoding the Glioblastoma Lactylation Landscape: Challenges and Emerging Avenues

Despite the existence of several studies that have provided preliminary insights into the mechanism of lactylation and its effects, there are still many challenges. Among them, three conspicuous aspects will be addressed in detail as follows. Firstly, the current assays for various lactylations are not standardized, which may affect the accuracy of the results. Secondly, in view of the complexity of the molecular pathways involved in lactylation, it is challenging to determine whether lactylation plays a primary regulatory role or is merely a secondary consequence of other metabolic processes, as evidenced by the current preliminary studies. Finally, based on the particularity of the structure and location of the central nervous system, coupled with the significant inter- and intratumor heterogeneity of glioblastomas, the existing in vitro models are limited in maintaining the cellular and mutational diversity of the parental tumors, resulting in the failure of the current targeted and monotherapeutic approaches to effectively curb disease progression [129]. We urgently look forward to further studies and discoveries to elucidate the exact molecular mechanisms underlying the role of lactylation in GBM and other cancers. At the same time, because the therapy strategy of lactate is still at the fundamental research stage, clinical trials should be designed to verify its effectiveness, so as to develop new diagnostic and therapeutic methods.

Currently, the progress of emerging technologies is facilitating further advances in lactylation research. The integration of genomics, transcriptomics, proteomics, metabolomics, and other multi-omics with single-cell sequencing has yielded promising applications in clear cell renal carcinoma (ccRCC) [130]. This is poised to elucidate the intricate relationship between lactylation and tumorigenesis and to offer a novel perspective on the precise treatment of GBM. Furthermore, combined with single-cell RNA sequencing and high-throughput screening technologies, we are expected to further reveal the functional differences in lactylation in different GBM subtypes, thus advancing the development of individualized treatment [131,132]. Therefore, we believe that in future, multi-omics sequencing technology, in conjunction with single-cell analysis, will be employed to develop organoid models of glioblastoma, which will facilitate a more accurate simulation of the complex microenvironment within GBM and enable the precise and specific targeted manipulation of lactylation levels, thereby allowing for a more detailed exploration of the causal relationship between lactylation levels and specific tumor phenotypes. Furthermore, this will establish a model for combining lactylation-targeting therapy and immunotherapy, which can provide insights into more targeted treatment strategies for patients.

## 6. Outlook for the Future Research Direction

Although the study of lactylation in GBM is still in the early stage of development, both opportunities and challenges exist simultaneously, which is undoubtedly an important clue to explore new therapeutic strategies. Future research should concentrate on the specific mechanism of lactylation in GBM cells to develop more efficient therapeutic targets, with the objective of transforming lactylation research from a fundamental to a clinical application, providing new strategies for the application of GBM in precision medicine with the aim of improving patient prognosis and quality of life.

## Abbreviations

GBM	glioblastoma
PTM	post-translational modification
HIF-1α	hypoxia-inducible factor 1 alpha
GLUT1	glucose transporter 1
TIGAR	the glycolytic and apoptotic regulator
mTOR	mammalian targets of rapamycin
TME	tumor microenvironment
MCTs	monocarboxylate transporters
GPCRs	G-protein-coupled receptors
HCAR1	hydroxycarboxylic acid receptor 1
CAFs	cancer-associated fibroblasts
TAMs	tumor-associated macrophages
Tregs	regulatory T cells
VEGF	vascular endothelial growth factor
Arg1	arginase 1
LDHA	lactate dehydrogenase A
APC/C	anaphase-promoting complex
LC-MS/MS	liquid chromatography–tandem mass spectrometry
LGSH	lactoyl-glutathione
EMT	epithelial–mesenchymal transition
cSCC	cutaneous squamous cell carcinoma
MMR	mismatch repair
DCA	dichloroacetate
Dex	dexmedetomidine
lactyl-CoA	lactyl-coenzyme A
HDAC	histone deacetylase
MDMs	monocyte-derived macrophages
CHC	α-cyano-4-hydroxycinnamate
